# Investigation of the Influence of Pre-Charged Hydrogen on Fracture Toughness of As-Received 2.25Cr1Mo0.25V Steel and Weld

**DOI:** 10.3390/ma11071068

**Published:** 2018-06-24

**Authors:** Yan Song, Mengyu Chai, Bin Yang, Zelin Han, Song Ai, Yilun Liu, Guangxu Cheng, Yun Li

**Affiliations:** 1 School of Chemical Engineering and Technology, Xi’an Jiaotong University, Xi’an 710049, China; songyan1211@mail.xjtu.edu.cn (Y.S.); chaimengyu929@stu.xjtu.edu.cn (M.C.); yangbinxjtu1993@163.com (B.Y.); hanzelin@stu.xjtu.edu.cn (Z.H.);; 2 School of Aerospace, Xi’an Jiaotong University, Xi’an 710049, China; ais@mail.dfstw.com (S.A.); yilunliu@mail.xjtu.edu.cn (Y.L.)

**Keywords:** welded joint, welding, hydrogen embrittlement, fracture toughness, 2.25Cr1Mo0.25V

## Abstract

Fracture failure caused by hydrogen embrittlement (HE) is a major concern for the system reliability and safety of hydrogen storage vessels, which are generally made of 2.25Cr1Mo0.25V steel. Thus, study of the influence of pre-charged hydrogen on fracture toughness of as-received 2.25Cr1Mo0.25V steel and weld is of significant importance. In the current work, the influence of hydrogen on fracture toughness of as-received 2.25Cr1Mo0.25V steel and weld was systematically studied. Base metal (BM) and weld metal (WM) specimens under both hydrogen-free and hydrogen-charged conditions were tested using three-point bending tests. Hydrogen was pre-charged inside specimens by the immersion charging method. The J-integral values were calculated for quantitatively evaluating the fracture toughness. In order to investigate the HE mechanisms, optical microscopy (OM) and scanning electron microscopy (SEM) were used to characterize the microstructure of BM and WM specimens. The results revealed that the presence of pre-charged hydrogen caused a significant decrease of the fracture toughness for both BM and WM specimens. Moreover, the pre-charged hydrogen led to a remarkable transition of fracture mode from ductile to brittle pattern in 2.25Cr1Mo0.25V steel.

## 1. Introduction

In the view of good resistance to corrosion and hydrogen damage, the low alloy chromium-molybdenum (Cr-Mo) steel has been extensively applied in the fabrication of high-pressure vessels such as hydrogen storage vessels in the petrochemical industry. In recent years, due to the increasing demand for higher efficiency of refining equipment, these vessels are required to be operated under higher pressure conditions. However, the Cr-Mo steel is not suitable to be applied in these extreme conditions. Therefore, a new vanadium (V)-modified Cr-Mo steel (i.e., 2.25Cr1Mo0.25V steel) that possesses better mechanical properties has been developed and employed to replace Cr-Mo steel in manufacture of hydrogen storage vessels [1,2].

In general, hydrogen storage vessels suffer a severe hydrogen environment with high pressure during the operating process. This may introduce hydrogen into the alloy steel, leading to a decrease in its mechanical properties, and this phenomenon is called hydrogen embrittlement (HE) [3]. This is considered to be one of the most critical issues that hinders the reliability of hydrogen storage vessels and causes fracture failure. Moreover, welded joints (for e.g., base metal (BM) and weld metal (WM)) widely exist in hydrogen storage vessels. These vessels usually operate under extreme work conditions (e.g., high pressure), which may easily lead to crack initiation, propagation, and even the fracture failure of welded joints. This can greatly compromise the safety of hydrogen storage vessels. Thus, the fracture toughness behavior of the welded joints has an essential meaning in assessing the reliability of hydrogen storage vessels [4,5]. Therefore, investigation on the influence of hydrogen on fracture toughness of welded joints and failure aspects of these joints due to HE has a significant contribution to the safety assessment and optimal design of hydrogen storage vessels. 

Other than the application of high-strength low-alloy 2.25Cr1Mo0.25V steel in fabrication of high pressure vessels, significant research efforts have been devoted to the study of potential properties and uses of 2.25Cr1Mo0.25V steel. For example, Pereira et al. [6] investigated the influence of hydrogen on 2.25Cr1Mo0.25V steel by tensile, mechanical, and hydrogen permeation tests. The results indicated that application of elastic stress on the hydrogenated samples resulted in the improvement of solubility of hydrogen and eventual increase of HE of this alloy. Guo et al. [7] studied the fracture toughness of advanced 9Cr and CrMoV steels by using the three-point bending tests. Furthermore, they studied the relationship between the microstructure and fracture toughness with the aid of an optical microscope, scanning electron microscope and transmission electron microscope. Gacia et al. [8] used the small punch test to investigate the influence of pre-charged hydrogen on the tensile mechanical properties of three different grades CrMoV steel. The results showed that three grades of CrMoV steel have different susceptibility to HE. Nonetheless, to our knowledge, influence of pre-charged hydrogen on fracture toughness of 2.25Cr1Mo0.25V welded joint has rarely been investigated. In our previous article [9], the influence of pre-charged hydrogen on the 2.25Cr1Mo0.25V steel and weld after post weld heat treatment (PWHT, i.e., Annealing) was studied. However, due to the influence of the PWHT, the reduction of fracture toughness caused by pre-charged hydrogen was limited, thus, it’s hard to clarify the intrinsic effect of pre-charged hydrogen on fracture toughness of 2.25Cr1Mo0.25V steel and weld. Investigating the intrinsic HE properties of 2.25Cr1Mo0.25V steel and weld, has a significant meaning on improving the HE resistance, such as the PWHT optimization. Therefore, to better understand the intrinsic hydrogen embrittlement (HE) properties, it is of great significance to investigate the influence of pre-charged hydrogen on the fracture toughness of 2.25Cr1Mo0.25V steel and weld without PWHT (as-received).

In this study, a series of as-received 2.25Cr1Mo0.25V steel and weld (i.e., without any PWHT was utilized). The three-point bending method was applied to test the fracture toughness J-integral values of BM and WM of as-received 2.25Cr1Mo0.25V welded joint. In order to study the influence of pre-charged hydrogen, the immersion charging method was carried out to pre-charge hydrogen into parts of BM and WM specimens. The surface morphology of fracture and the microstructures of BM and WM specimens were investigated by means of scanning electron microscopy (SEM) and optical microscopy (OM), respectively, to clarify the mechanisms of HE.

## 2. Experimental Details

**Figure 1:**
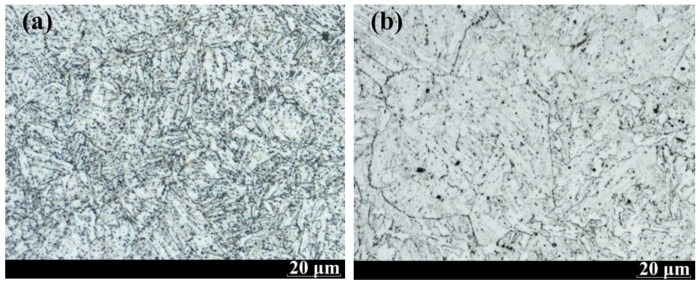
Optical microscopy (OM) images of 2.25Cr1Mo0.25V steel and weld: (**a**) base metal (BM) and (**b**) weld metal (WM).

The 2.25Cr1Mo0.25V steel plates, with dimension of 800 mm length, 320 mm width, and 98 mm thickness, were provided by ArcelorMittal Company (Luxembourg City, Luxembourg). The 2.25Cr1Mo0.25V steel plates were joined through the method of the narrow-gap welding, which was performed by Lanzhou LS Heavy Equipment Co., Ltd (Lanzhou, China). Welding details are as follows: current, 500 A; voltage, 32 V; and travel speed, 22 mm·min^−1^. To avoid the effect of PWHT on experimental results, no PWHT was performed after welding process. Prior to the machining of specimens from the welded joint, the joints were examined by radiographic testing using X-rays to locate defects. Figure 1 presents the typical microstructures of BM and WM of 2.25Cr1Mo0.25V steel welded joint, clearly exhibiting the existence of bainitic microstructures in both the cases. However, the grain size of WM is larger than that of BM. The chemical composition of 2.25Cr1Mo0.25V welded joint (i.e., BM and WM) is provided in Table 1.

**Figure 2:**
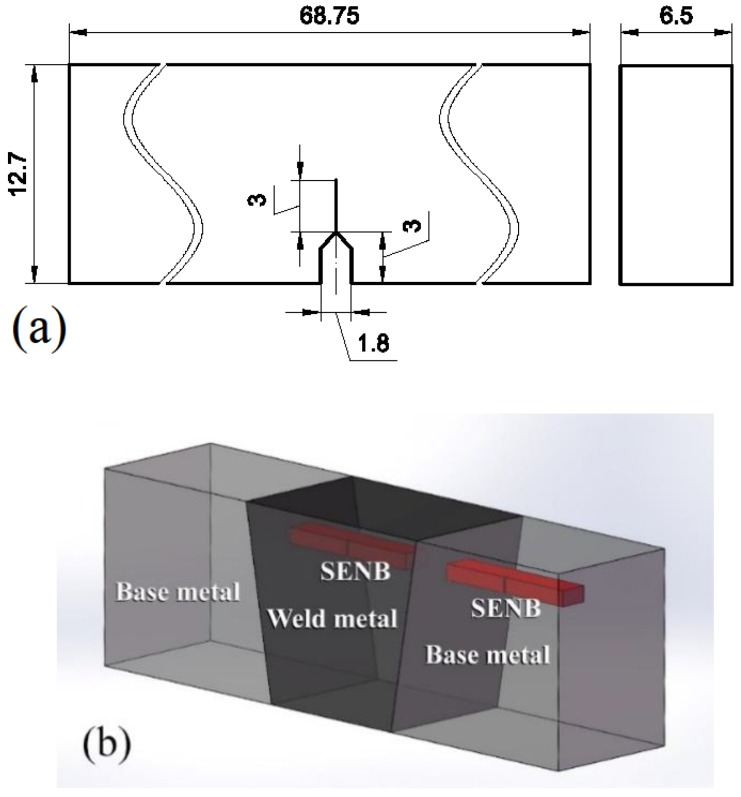
Single edge notched-bend (SENB) specimens: (**a**) Dimension of BM and WM three points specimen (units: mm); (**b**) Schematic of positions of the SENB specimens’ sampling.

Based on ISO 12135 standard [10], the BM and WM single edge notched-bend (SENB) specimens were machined from 2.25Cr1Mo0.25V welded joint. Figure 2 exhibits the geometric dimensions and positions of the SENB specimens. The SENB specimens for Base Metal (BM) and Weld Metal (WM) were machined near the top surface of the welded joint. The notch of BM and WM is in the Base Metal and Weld Metal, respectively. The specimens were obtained with the weld longitudinal with respect of the pre-crack. All specimens were ground sequentially with 1500 grit abrasive paper and mirror polished. using 1 μm diamond paste. After the grinding process, the specimens were cleaned with de-ionized water and methanol, and then dried with the help of a stream of cold air. Finally, the specimens were corroded using a 5% Nital solution and immediately cleaned with methanol. Metallurgical microscopy was employed to observe metallographic structure of BM and WM.

In this study, hydrogen was charged by immersing the specimens in an aqueous solution of ammonium thiocyanate (NH_4_SCN, 20 mass%, pH 4.8) at room temperature. The NH_4_SCN immersion charging method has been proved to be simpler and more efficient compared to other hydrogen charging methods, such as the electrochemical charging method [11]. Moreover, it has also been previously employed for HE studies of several alloys involving the high strength Cr-Mo steels [11,12]. In the present investigation, specimens were initially pre-cracked under sinusoidal cyclic load to gain a 3 mm fatigue crack, wherein the maximum and minimum peak forces were 4 kN and 0.4 kN, respectively [13]. Subsequently, half the number of the pre-cracked specimens was immersed in NH_4_SCN solution mentioned above for 96 h. Then, the specimens were rinsed, dried and immediately tested within 15 min. 

The three-points bending experiments were performed using a mechanical testing machine (MTS 880/25t; MTS Systems Corporation, Eden Prairie, MN, USA) at room temperature. The speed of loading was 0.5 mm·min^−1^. The curves of load-displacement were obtained to estimate J-integral values. In order to investigate the fracture appearances, fatigue crack growth processes were further carried out on the tested specimens to facilitate fatigue crack formation. Fracture morphology of the specimens was observed using the SEM (SU 3500; Hitachi, Tokyo, Japan).

In this study, the J-integral was used for the assessment of fracture toughness. Where, the J-integral consists of the elastic and plastic parts and it can be expressed as follows:

(1) $$J=J_{el}+J_{pl}$$

where the elastic part is shown as follows:

(2) $$J_{el}=\frac{K^{2}\left(1-\nu^{2}\right)}{E}$$

where, the K represents the stress intensity factor and can be calculated by:

(3) $$K=\left(\frac{PS}{BW^{3/2}}\right)f\left(a_{0}/W\right)$$

where P, S, B and W respresent the maximum load, the span, the thickness and width of the specimens, respectively. ν denotes the Poisson’s ratio and with a value of 0.3, E represents the Young’s modulus and with a value of 210 GPa, $a_{0}$ is the initial crack length, and $f\left(a_{0}/W\right)$ denotes the coefficient of stress intensity factor. On the other hand, the plastic part of J integral is estimated by:

(4) $$\;J_{pl}=\frac{2A_{pl}}{B\left(W-a_{0}\right)}$$

where $A_{pl}$ represents plastic part of deformation energy. According to ISO 12135 standard, the J-integral in this study represents the fracture resistance at the maximum load.

## 3. Results and Discussion

### 3.1. Estimation of Fracture Toughness

**Figure 3:**
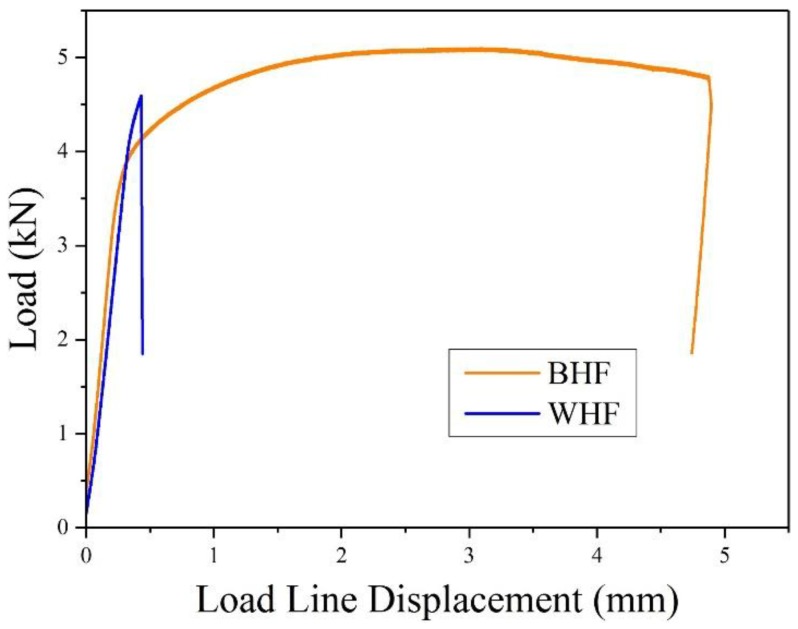
The variation of load with respect to the displacement for hydrogen free specimens.

Figure 3 displays the typical load-displacement curves for the BM and WM specimens without hydrogen charged condition (BHF and WHF, respectively). Apparently, there exists remarkable differences in their mechanical properties. First, an obvious two-stage characteristic process consisting of the elastic and plastic deformation stages is observed in the BM specimen, whereas only elastic deformation stage is shown for the WM specimen. Furthermore, for the BM specimen, before the final drop of load, a prominent load plateau is evidently seen because of the significant plasticity-induced strain-hardening phenomenon and the blunting of newly generated crack tip. However, this figure clearly shows that for the WM specimen, load plateau is not observed, and the load suddenly drops down after the elastic deformation stage. Such pronounced difference revealed that BM possessed significantly superior toughness compared to the WM specimen. In addition, the maximum load of the BM specimen was obtained to be higher than that of the WM specimen. Thus, the coexistence of prominent plasticity and higher load in the BM specimen indicated superior fracture toughness to that of the WM specimen.

**Figure 4:**
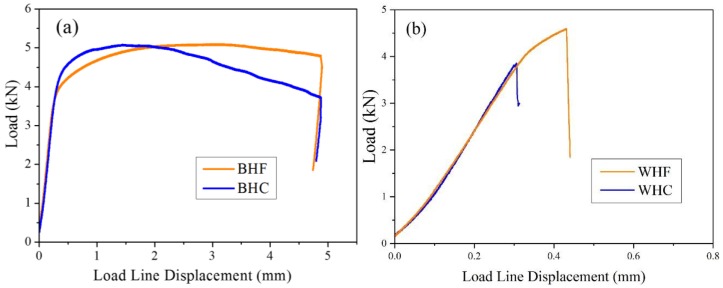
The variation of load with respect to the displacement. (**a**) and (**b**) represent BM and WM specimens under hydrogen-free (HF) and hydrogen-charged (HC) conditions.

Figure 4 displays the typical load-displacement curves for the BM and WM specimens under both hydrogen-free (HF) and hydrogen-charged (HC) conditions. On one hand, for BM specimens, similar load line displacements can be seen under both HF and HC conditions, suggesting the similar stable crack propagation distances. However, a less pronounced load plateau is observed under the HC condition. The load reaches the maximum value with only a minor displacement, and subsequently decreases continuously. On the other hand, WM specimens show the only occurrence of elastic deformation stages. However, a decreased maximum load is found under the HC condition compared to that under the HF condition. Consequently, the fracture toughness of both base metal and weld metal specimens under the HF condition was found to be superior to that under the HC condition. The detrimental influence of pre-charged hydrogen on fracture toughness could be clarified based on the fracture morphology, which is discussed in the following section.

**Figure 5:**
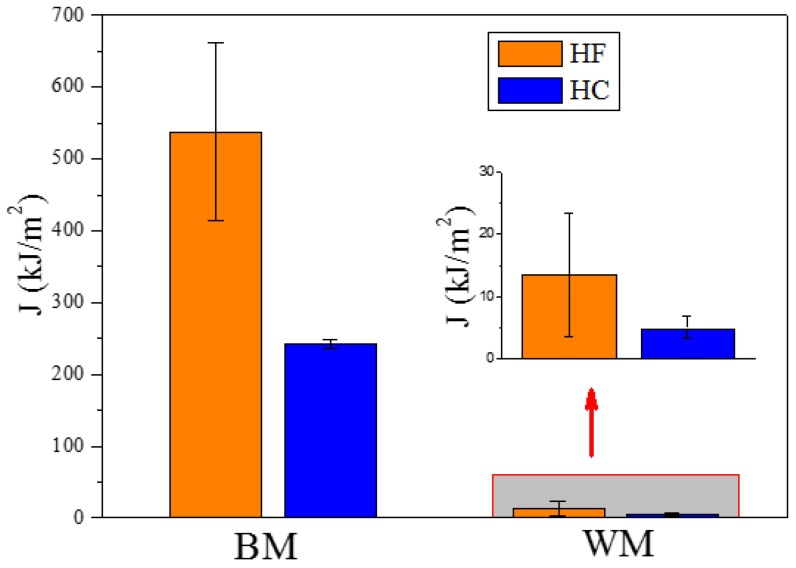
J-integral values of BM and WM specimens under both HF and HC conditions. The red arrow points to the magnification of the fracture toughness values of WM.

Figure 5 exhibits the results of J-integral. The average J-integral values of BM and WM specimens in the condition of HF were calculated to be 538.1 and 13.6 kJ·m^−2^, respectively. According to literature, the critical fracture toughness $J_{IC}$ calculated by ISO 12135 standard for 2.25Cr1Mo0.25V steel was 555 kJ·m^−2^ [14], which was in accordance with results in this work. The very low fracture toughness of WM was attributed to the absence of plastic deformation stage in load-displacement curve. In addition, notably, the pre-charged hydrogen contributed to a remarkable decrease of the fracture toughness. The average J-integral of base metal specimen under the HC condition decreased to 242.3 kJ·m^−2^; however, the value of weld metal specimen reduced to 5.1 kJ·m^−2^, despite the absence of plasticity. In order to describe the severity of HE, the sensitivity to hydrogen $\delta_{H}$ can be expressed as follows:

(5) $$\delta_{H}=\frac{J-J_{H}}{J}\times 100\%$$

where J and $J_{H}$ represent average J-integral values gained from fracture toughness tests under HF and HC conditions, respectively. The $\delta_{H}$ values were estimated to be 54.9% and 62.5% for BM and WM specimens, respectively. These significant values indicated that the pre-charged hydrogen caused serious HE in both BM and WM of 2.25Cr1Mo0.25V steel and weld, causing a remarkable decrease in fracture toughness and may ultimately leading to HE failures of hydrogen storage vessels. Therefore, significant attention should be paid to the structural health monitoring on hydrogen-contacting equipment to avoid HE failures during the operation and maintenance of equipment due to the high sensitivity of 2.25Cr1Mo0.25V steel and weld to hydrogen.

### 3.2. Fracture Appearance

**Figure 6:**
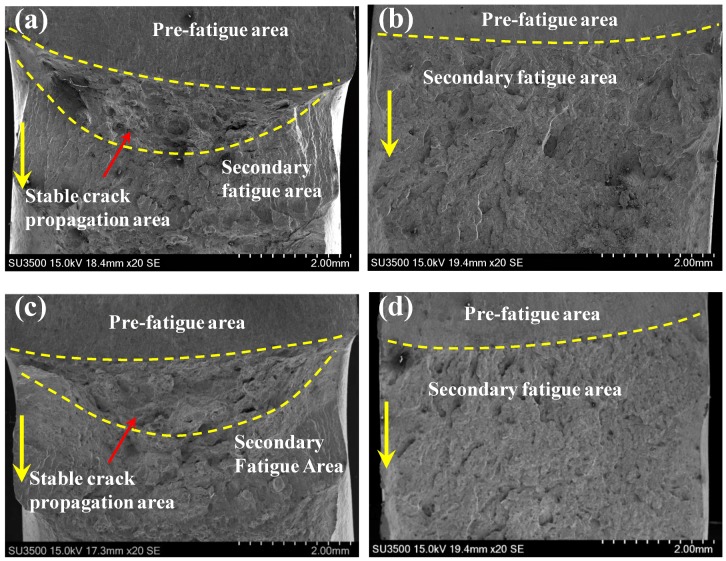
The macroscopic fracture surface. HF condition: (**a**) BM and (**b**) WM. HC condition: (**c**) BM and (**d**) WM. The yellow dash lines differentiate the different areas on fracture surface, and the yellow arrows mark the direction of crack propagation.

To provide complete insight into HE mechanisms, the fracture appearance of BM and WM specimens under both HF and HC conditions was investigated by SEM. Figure 6 exhibits the macroscopic fracture surfaces of base metal and weld metal specimens under different conditions. Obviously, the fracture surfaces of BM specimens include three distinct regions, that is, the pre-fatigue area, the stable crack propagation area, and the secondary fatigue area, as shown in Figure 6a and c. However, no evident stable crack propagation region is found on the fracture surfaces of WM specimens, and the interfaces between the stable crack propagation area and secondary fatigue area are indistinct. This result is consistent with the above-mentioned load-displacement results, indicating completely brittle features of the WM specimen.

**Figure 7:**
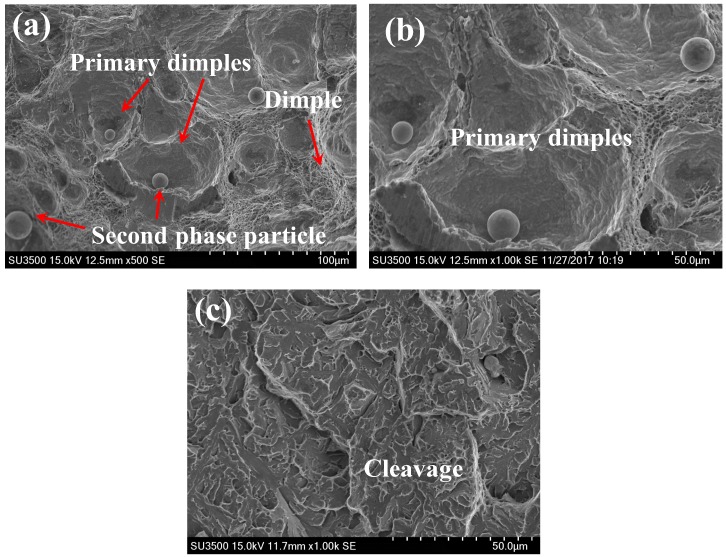
The microscopic fracture surface under HF condition: (**a**, **b**) BM and (**c**) WM. (**b**) is the magnification of primary dimples in (**a**).

Figure 7 shows the detailed fracture appearance of BM and WM specimens under the HF condition. Figure 7a clearly demonstrates that fracture surface of base metal specimen is composed of numerous large primary dimples with diameters of 10–50 μm and a number of fine dimples. Figure 7b indicates the magnification of primary dimples in Figure 7a. Such remarkable features apparently suggest that the fracture micro-mechanism of BM is ductile fracture, which is predominantly governed by micro-voids nucleation, growth and coalescence [15]. Moreover, some second phase particles and inclusions (5–20 μm in diameter) are also located in the center of primary dimples, indicating that the dimples were liable to form from the second phase particles and inclusions. On the other hand, Figure 7c exhibits the existence of the cleavage fracture feature on the fracture surface of WM specimen, indicating completely brittle fracture. Previous studies have demonstrated that in high strength steel, more plastic energy will be consumed so as to generate larger sized voids [7]. Therefore, the appearance of ductile dimples contributed to superior fracture toughness in BM. 

**Figure 8:**
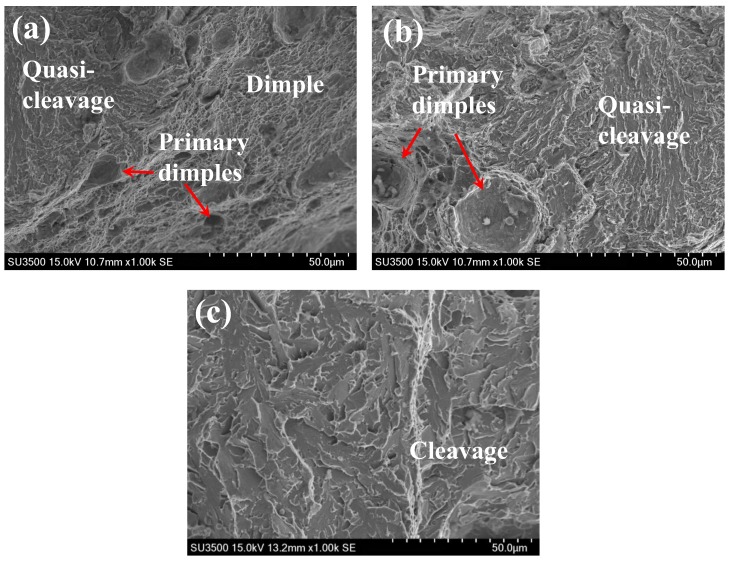
The microscopic fracture surface under HC condition: (**a**, **b**) BM and (**c**) WM.

Figure 8a and b demonstrate that compared to the HF condition, the detailed fracture morphology of BM specimen under the HC condition shows a mixed ductile and brittle feature. Figure 8a exhibits two different regions, namely, the quasi-cleavage region with river line pattern and ductile regions containing shallow dimples and small-sized primary dimples. Figure 8b shows ductile feature with primary dimples and more prominent quasi-cleavage fracture feature with river lines. The size of primary dimples mainly ranges from 4 to 25 μm, which are significantly smaller compared to those under the HF condition. The reduced size of primary dimples indicated a lower resistance to crack propagation as less dissipated energy was required for generating dimples with small size. Moreover, the appearance of quasi-cleavage regions indicated a major brittle feature, indicating the occurrence of a local quasi-cleavage due to the presence of hydrogen. In contrast, Figure 8c demonstrates that the WM specimen under the HC condition shows a complete cleavage fracture feature because of the absence of the plastic deformation stage. Therefore, the coexistence of brittle regions and the dimples with decreased size contributed to a prominent decrease of fracture toughness of BM specimen under the HC condition. The fracture toughness J-integral values, SEM features and fracture mechanisms are summarized in Table 2.

### 3.3. Mechanisms of HE

HE, serving as a process by which metallic materials such as steel become brittle and fracture due to the introduction and subsequent diffusion of hydrogen, results in the degradation of the mechanical properties of metal such as loss of ductility and strength. This generally leads to the reduction of fracture resistance and cracking from the hydrogen concentrated region [16]. In this study, the pre-charged hydrogen caused a remarkable decrease of fracture toughness for both BM and WM specimens. Moreover, the pre-charged hydrogen contributed to a prominent transition of fracture mode from ductile to brittle pattern, which might lead to unpredictable catastrophic failures of hydrogen-contacting equipment. Despite that there is a strong agreement on the harmful influence of hydrogen on the mechanical properties of various alloys, the mechanism that contributed to such damage and premature failure is still being debated [17], because HE is a very complicated process with many underlying mechanisms. Therefore, in the present work, in-depth systematic explorations were carried out to investigate the HE mechanisms. Since the WM specimens under both HF and HC conditions show complete cleavage fractures, the SEM analyses of WM could not provide enough explanation of its HE mechanism. Thus, the following analyses based on SEM were mainly carried out to investigate the HE mechanism of BM.

Often, HE failure results from a combination of several influences, making the determination of governing mechanism extremely difficult. Therefore, in the past decades, several mechanisms have been proposed to describe HE in non-hydride forming materials. The two well-developed HE mechanisms are the hydrogen-enhanced decohesion (HEDE) mechanism and the hydrogen-enhanced localized plasticity (HELP) mechanism. HEDE mechanism hypothesizes a reduction in the cohesive bond energy between atoms (such as iron atoms) in the presence of hydrogen [18]. It is based on the increased solubility of hydrogen in a tensile strength field. This leads to brittle crack propagation under highly concentrated stress. The brittle fracture features such as intergranular and cleavage fracture can support the HEDE mechanism, even though there is no available direct measurement of cohesive bond energy at present [19]. On the other hand, the HELP mechanism indicates that presence of hydrogen will strengthen the mobility of dislocations by weakening strength of barriers to dislocations motion [20]. This promotes some macroscopic failures through highly localized plastic deformation and ductile rupture processes. This mechanism has attained substantial support and can account for the localized plastic deformation observed on fracture surfaces in the case of brittle fracture of specimens [16,21,22]. In this study, the occurrence of localized ductile and brittle characteristics in the fracture surface of HC specimens clearly shows that the HEDE mechanism and HELP mechanism both existed in 2.25Cr1Mo0.25V steel. It is worth noting that the 96 h hydrogen pre-charging process resulted in a critical concentration of trapped hydrogen in the large areas of bainitic grains. The concentrated further hydrogen led to the reduction in the cohesive energy of metal atoms in grains, decrease in the fracture initiation stress, and consequently promoted the quasi-cleavage fracture, as shown in Figure 8a and b. It is noteworthy that the HEDE mechanism could account for the appearance of brittle fracture. However, the HELP mechanism contributed to characteristics of ductile dimple fracture with micro-void nucleation, growth, and coalescence, as shown in Figure 8a and b. The pre-charged hydrogen might decrease the critical stress of void nucleation. The decrease of formation energy of vacancy and free surface could accelerate the void nucleation [23]. This explains the formation of primary dimples with reduced size and shallow fine dimples in the fracture surfaces under the HC condition. Thus, one could conclude that the simultaneous action of the HEDE mechanism and HELP mechanism caused the significant decline in fracture toughness of 2.25Cr1Mo0.25V steel. 

## 4. Conclusions

In this study, the influence of pre-charged hydrogen on fracture toughness of as-received 2.25Cr1Mo0.25V steel and weld was researched. Three-point bending experiments were performed on base metal (BM) and welded metal (WM) specimens under both hydrogen-free (HF) and hydrogen-charged (HC) conditions. The J-integral values were calculated for the quantitative assessment of fracture toughness. Moreover, the HE mechanisms were studied to gain an insight into the fracture mechanism of 2.25Cr1Mo0.25V steel in the presence of pre-charged hydrogen. The main conclusions of this study are listed as:

1. The fracture toughness of the BM specimen was much higher than that of the WM specimen in 2.25Cr1Mo0.25V welded joints. Furthermore, the fracture mode in the BM specimen was ductile, whereas it was a completely brittle fracture in the WM specimen.

2. The presence of pre-charged hydrogen caused a remarkable decrease of the fracture toughness for both BM and WM specimens. Moreover, the action of hydrogen caused a prominent transition of fracture mode from ductile to brittle pattern in 2.25Cr1Mo0.25V steel. It was believed that the simultaneous action of the HEDE and HELP mechanisms accounted for the remarkable decrease in fracture toughness of hydrogen pre-charged 2.25Cr1Mo0.25V steel.

## Figures and Tables

**Table 1:** Chemical composition of BM and WM (wt.%).

Element	C	Si	Mn	P	S	Cr	Mo	V	Al	Ni	Cu
BM	0.15	0.1	0.54	0.009	0.01	2.3	0.98	0.3	0.05	-	-
WM	0.12	0.22	1.07	0.004	0.004	2.45	1.03	0.42	-	0.03	0.11

**Table 2:** Summary of results for the microscopic fracture surface for BM and WM specimens under HF and HC conditions.

Specimen	Hydrogen Condition	Fracture Toughness (kJ·m^−2^)	SEM Features	Fracture Mechanisms
BM	HF	538.06 ± 124.32	dimples	ductile fracture
BM	HC	242.27 ± 6.83	dimples and quasi-cleavage facets	ductile and brittle fracture
WM	HF	13.55 ± 9.85	cleavage facets	brittle fracture
WM	HC	5.06 ± 1.77	cleavage facets	brittle fracture
